# The Effect of High-Intensity Ultrasound on the Physicochemical and Microbiological Properties of Mexican Panela Cheese

**DOI:** 10.3390/foods9030313

**Published:** 2020-03-09

**Authors:** Luis M. Carrillo-Lopez, Monica G. Juarez-Morales, Ivan A. Garcia-Galicia, Alma D. Alarcon-Rojo, Mariana Huerta-Jimenez

**Affiliations:** 1 Faculty of Animal Science and Ecology, Autonomous University of Chihuahua, Perif. Francisco R. Almada km 1, Chihuahua, Chih 31453, Mexico; 2 National Council of Science and Technology, Av. Insurgentes Sur 1582, Col. Crédito Constructor, Del. Benito Juárez, Ciudad de México 03940, Mexico

**Keywords:** high-intensity ultrasound, fresh cheese, microbiological control, nutritional quality

## Abstract

High-intensity ultrasound could be an alternative to pasteurization for cheeses made with fresh raw milk, the properties of which must be preserved as part of their intangible cultural heritage, such as Panela cheese in Mexico. This research aimed to study the effect of the amplitude (50% and 100%) and application time (0, 5, and 10 min) of ultrasound treatment of fresh raw milk, on the yield and microbiological and physicochemical qualities of Panela cheese after 24 h of storage at 4 °C. The yield was increased to 24.29% with 10 min of ultrasonication, although the amount of exudate was higher in the ultrasonic product than in the control (20.33%). As the ultrasonication time increased, the yellowness (b*) increased significantly, while the hue angle decreased (with values close to 90°), resulting in evident yellow tones in cheeses made with milk treated for 10 min. The pH significantly increased from 6.6 to 6.74 with 5 min of ultrasound, but decreased to 6.37 with 10 min of ultrasonication. Although no significant differences were found in fat content, the protein significantly increased with 5 min of sonication, but it decreased markedly when ultrasound was applied for 10 min. Ultrasound treatment with amplitudes of 50% effectively decreased the counts of coliform bacteria regardless of ultrasonication time. However, the mesophilic bacteria increased by a 0.9 log with an amplitude of 100% and 10 min treatment. The results showed that ultrasound improved the yield and microbial, nutritional, and physicochemical properties of Panela cheese.

## 1. Introduction

Ultrasound technology applied in the food industry is classified into two types: low-intensity (LIU) and high-intensity ultrasound (HIU). LIU does not cause physicochemical, biochemical, or microstructural modifications in the food, since it uses power lower than 1 W cm^−2^ and frequencies greater than 100 kHz [1]. HIU produces changes in food because it uses power greater than 10 W cm^−2^ and low frequencies in the range of 20 to 100 kHz [2]. The acoustic energy of the transducers (both bath equipment and ultrasonic processors) causes vibrations in the molecules that constitute the food. The compression and rarefaction cycles of the sound waves in the liquid produce cavities that grow when they produce bubbles (acoustic cavitation), which then collapse, generating temperatures of 5000 K and pressures of 100 Mpa [2,3]. Factors such as viscosity, temperature, pressure, and ultrasonic frequency and intensity affect the ultrasonication process. Bubble collapse is more violent with high temperatures and low viscosities in the liquid [4]. Ultrasound is a technology that produces structural changes that modify the integrity of fat cells and intensifies fermentation processes, producing flavoring compounds, so it has potential applications in cheese processing [5]. However, several researchers have also observed other effects such as a decrease in viscosity in concentrated milk [6]; improvement in functional properties of proteins, such as gelation during cheese making and solubility in recombined products [7]; denaturation of whey proteins [8]; and inactivation of pathogenic bacteria such as *Escherichia coli* or deteriorating agents such as *Saccharomyces cerevisiae* [9]. A potential application of HIU in cheesemaking is the alteration of casein micelles with smaller particle sizes to make the curdling more efficient [10]. In this regard, studies have been inconclusive. Some researchers have found a reduction in micelle size, greater denaturation of serum proteins, and better gel firmness [11,12,13]. Others have shown a delay in serum separation and an increase in viscosity [14], as well as an improvement in the gel set time and firmness when the pH of milk is modified to 8.0 before the application of HIU [12,15]. Liu et al. [12] studied the effects of HIU on skim milk made by reconstitution of skim milk powder, with a treatment of 20 kHz at 30 °C. They found an improvement in the gelatinization and firmness of the curd when the pH of the ultrasound-treated milk was 8 and was re-adjusted back to 6.7. These superior renneting properties were attributed to the reduction in the size of particles in milk and possible changes to the protein hydrophobicity caused by the physical effects of cavitation when applying low-frequency ultrasound. The present study showed an increase in the yield of cheese using 5 and 10 min ultrasonication of raw milk with a pH of 6.7, but there were not enough data to assume an effect of protein changes.

Regarding microbiological counts, reductions have been reported of up to 3 log for aerobic bacteria and lactic acid bacteria [16], while aerobic bacteria can be reduced up to 99% [17]. The differences between the reported results are probably due to different HIU parameters used (time, frequency, intensity, amplitude, temperature, whole and/or skim/recombined milk, ultrasound system, etc.).

In Mexico, just over 30 genuine artisan cheeses have been described. Among them, Panela cheese is widely distributed in the national market. It is a fresh soft cheese, self-pressed and made with pasteurized cow’s milk [18,19]. Panela cheese is made with fresh raw milk in many regions of the country, and experiences very early swelling due to the activity of coliform microflora. These microorganisms produce gases by fermentation, causing holes in the paste, and could include pathogenic bacteria [18]. The most important step in cheesemaking is the formation of curd (gel). Curd firmness depends on factors such as temperature, enzyme concentration (rennet), and microbial load [20]. Developments in cheese technology seek to reduce the time and increase the yield, which normally ranges between 10% and 12% depending on the type of cheese. Hence, new technologies such as HIU must be implemented to improve such aspects without detracting from quality. The positive effect of HIU on milk curdling and control of microorganisms could notably improve the quality of cheeses made with fresh raw milk. In this research, a factorial experiment was designed to evaluate the effects of the amplitude and time of HIU on raw cow milk, and its impact on the yield and physicochemical and microbiological qualities of Panela cheese.

## 2. Materials and Methods

### 2.1. Treatment Design and High-Intensity Ultrasound (HIU)

Fresh raw cow milk was obtained from the Dairy Production Units of the Faculty of Animal Science and Ecology of the Autonomous University of Chihuahua, Mexico. The milk came from healthy, well-fed Holstein cows and it was received at 4 °C, 4 h after milking. The raw milk was treated with HIU. Before the application of the treatments, the physicochemical quality of the fresh raw milk was analyzed using a LactoScan LW Milk Analyzer (Milkotronic Ltd.^®^, Nova Zagora, Bulgaria). Measured milk parameters were titratable acidity, fat, protein, lactose, and non-fatty solids (SNG) (Table 1). This study was designed as an experiment with two completely random factors: time of ultrasonic treatment (0, 5, and 10 min) and amplitude of HIU (50% and 100%, 24 kHz, 400 W in continuous mode). Previous tests with different times and amplitudes of HIU applied (ultrasonic processor Hielscher UP400s) to fresh raw milk resulted in bitter and rancid flavors when treated for more than 10 min (results not shown). Therefore, the treatments evaluated were 0 min/50%, 5 min/50%, 10 min/50%, 0 min/100%, 5 min/100%, and 10 min/100% (time/amplitude). The maximum temperature reached during the ultrasonication process was 16 °C. Once the milk was ultrasonicated according to the evaluated treatments, Panela cheese was produced.

### 2.2. Panela Cheesemaking

**Figure 1:**
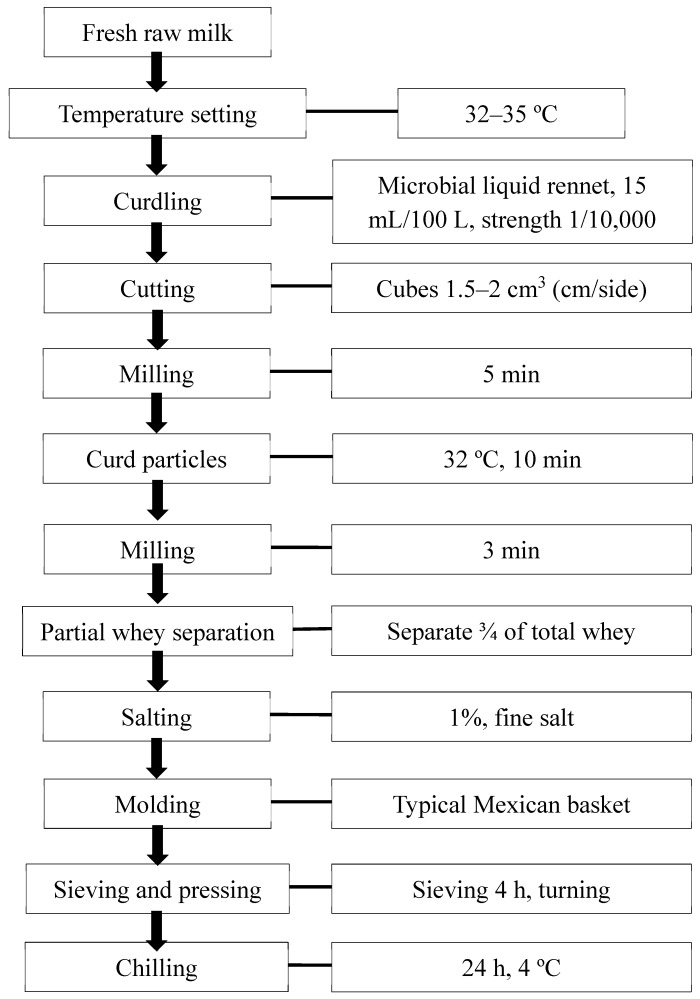
Flow chart of Panela cheese production.

Panela cheese was made following the methodology of Villegas [18], using Cuamix^®^ (CHR Hansen A/S, Hørsholm, Denmark) microbial rennet. The procedure is described in Figure 1. The total volume of milk used to make Panela cheese was 2.5 L per treatment. The manufacturing process was as follows. Initially, the temperature of the fresh raw milk was increased by heating to 32–35 °C, and rennet previously diluted in 30 mL of water was added and gently stirred to disperse it into the milk. The cheese milk was left for 30 to 50 min to form curds. Subsequently, the cheese curd was horizontally and vertically cut, using long blunt knives, into 1.5–2 cm^3^ cubes. The cubes were allowed to settle for 5 min, and then they were gently shaken with a wooden shovel for 10 min and left to settle for 3 more min to promote the release of approximately ¾ of the cheese whey. Fine salt (1% of the initial milk volume) was added and stirred, the curd was milled in a ribbon-shaped plastic basket, and salt was mixed into it to arrest acid development. The cheese was left to drain for 4 h, being turned every 30 min to promote syneresis. The pressed blocks of cheese were then removed from the basket and packed in plastic bags to be stored at 4 °C for 24 h. At this point, the cheese was ready to be consumed or marketed.

### 2.3. Actual and Exudate Cheese Yield

The actual yield of Panela cheese was estimated after 24 h of storage at 4 °C, considering the weight of the cheese obtained in relation to the weight of the processed milk (volume × density). The yield was expressed in kg of cheese/100 L of milk. The amount of exudate during storage was determined by measuring the volume of exudate in the vacuum package after 24 h of storage at 4 °C.

### 2.4. Microbiological Evaluation

The count (CFU/mL) of mesophilic bacteria, psychrophilic bacteria, and total coliforms of Panela cheese was performed after 24 h of storage (4 °C) to ensure the safety of the cheese. Sterile diluent (0.1% peptone) was used to prepare dilutions from 10^−1^ to 10^−5^ for microbiological analyses. Mesophilic and psychrophilic bacteria were inoculated onto sterile plates prepared with standard count agar (PCA, CM0325; Oxoid) and incubated at 35 ± 2 °C for 48 ± 2 h and at 5 ± 2 °C for 168 h, respectively. Total coliforms were quantified on violet red bile glucose agar (VRBG, CM0325; Oxoid). The agar plates were placed in an anaerobic jar and incubated at 35 ± 2 °C for 48 ± 2 h. To calculate the colony-forming units (CFU/mL), the number of colonies was multiplied by the dilution factor. The results in CFU/mL were transformed logarithmically (log_10_) before statistical analysis.

### 2.5. Physicochemical Characteristics

The moisture [21], protein [22], and fat [23] content in the cheese was determined 24 h after production. The color space was determined using the CIE (Commission Internationale de lÉclairage) parameters L*, a*, and b*, where L* is luminosity, a* (+) is the redness, and b* (+) expresses the yellowness. The measurements were obtained with a colorimeter (Konica Minolta, CR 400, Tokyo, Japan) according to the CIE reference system (Commission Internationale de lÉclairage). Three readings were taken for each sample in different areas and the averages for the values of L*, a*, and b* were taken; chroma (C*) and angle hue (H*) were calculated by means of the expressions C* = √a*^2^ + b*^2^ and H* = tan^−1^ (b*/a*), respectively.

The pH was measured with a pH meter (Sentron, Model 1001, Woonsocket, RI, USA). The measurements were taken directly on the cheese. The electrode was inserted into the cheese at a depth of 2 cm. Three readings were taken in three different areas of the sample and the average was obtained.

### 2.6. Data Analyses

The microbiological counts expressed in CFU/mL were transformed into logarithmic units in base 10 for the analysis of the results. Data were analyzed in a completely randomized factorial design with the SAS 9.4 TS Level 1M1 program (SAS Institute Inc., Cary, NC, USA). An experiment with a completely randomized factorial design with two factors was used. The first factor was the ultrasound treatment time (0, 5, and 10 min), and the second factor was the ultrasound amplitude (0% and 50%), resulting in a full factorial experiment with two controls, 0 min HIU/50% amplitude and 0 min/100% amplitude.

## 3. Results and Discussion

### 3.1. Actual Yield and Exudate of Cheese

**Figure 2:**
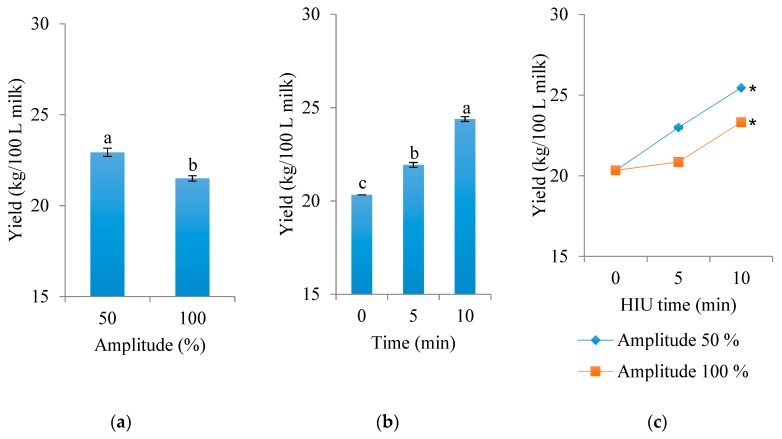
Effect of high-intensity ultrasound (HIU) on the yield of Panela cheese made with raw milk treated and stored for 24 h at 4 °C. (**a**) Effect of amplitude (mean of the two ultrasound times), (**b**) effect of time (mean of the two amplitudes), (**c**) interaction of both factors (time and amplitude). Asterisk (*) shows significant difference (*p* < 0.05) between treatments. ^a,b,c^ Different letters indicate significant differences between treatments (*p* < 0.05).

Significant differences by effect of ultrasonic amplitude (*p* < 0.0001) and ultrasonic time (*p* < 0.0001) of fresh raw milk were found in the yield of Panela cheese after 24 h at 4 °C (Figure 2a,b, respectively). The highest yield (25.46 kg cheese/100 L milk) was obtained when fresh raw milk was sonicated at an amplitude of 50% for 10 min. The lowest yields were obtained by using untreated fresh raw milk (control, 0 min of HIU, 20.33 kg cheese/100 L milk) and when the milk was treated with an amplitude of 100% (22.09 kg cheese/100 L milk). Therefore, times of 0 and 5 min of ultrasonication treatment of fresh raw milk significantly lowered the Panela cheese yield.

Liu et al. [12] studied the effects of HIU on skim milk made by reconstitution of skim milk powder, with a treatment of 20 kHz and 30 °C. They found an improvement in the gelatinization and firmness of the curd, when the pH of the ultrasonicated milk was 8 and re-adjusted back to 6.7. These superior renneting properties were attributed to a reduction in the size of particles in the milk and possible changes to the protein hydrophobicity caused by the physical effects of cavitation when applying low-frequency ultrasound. The present study showed an increase in the yield of cheese using 5 and 10 min ultrasonication of raw milk with a pH of 6.7, but there were not enough data to assume an effect of protein changes. However, Bermúdez-Aguirre, Mobbs, and Barbosa-Cánovas [20] also found an increase in the yield of fresh cheeses made with ultrasonicated milk. According to Villamiel et al. [24], ultrasound accelerates curd hardening, leading to a final product with higher firmness via the activity of chymosin, pepsin, and other proteolytic enzymes.

**Figure 3:**
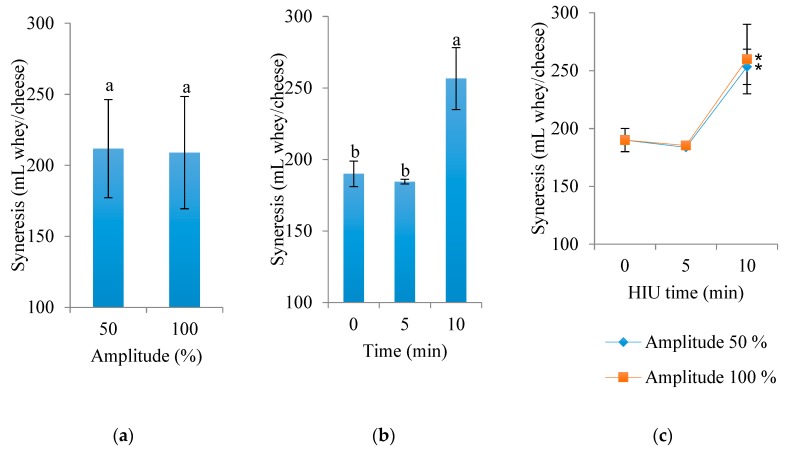
Effect of HIU on the syneresis of Panela cheese made with raw milk and stored for 24 h at 4 °C. (**a**) Effect of amplitude (mean of the two ultrasound times), (**b**) effect of time (mean of the two amplitudes), (**c**) interaction of both factors (time and amplitude). Asterisk (*) shows significant difference (*p* < 0.05) between treatments. ^a,b^ Different letters indicate significant differences between treatments (*p* < 0.05).

Regarding the volume of syneresis of Panela cheese after 24 h at 4 °C (Figure 3), significant differences were found due to the effect of HIU treatment time (*p* < 0.0001). The amplitude of HIU in raw milk had no effect on the volume of syneresis (*p* = 0.7001). However, the interaction of both factors (time and HIU amplitude) was significant (*p* < 0.0001) for the volume of separated whey. Cheese made with raw milk treated with HIU for a long time (10 min) released a higher amount of whey during storage (256.6 mL), while cheese treated with 0 and 5 min HIU presented less whey syneresis (190 and 184.5 mL, respectively).

Interestingly, the treatments with the highest yield (10 min HIU) were those that showed the highest syneresis during storage. So, there is a possibility that the phosphocasein matrix in these treatments was able to retain a higher volume of whey, slowing the process of syneresis without decreasing the solid content, but no measurements related to this characteristics were performed in the present study. Fresh cheeses are typical in Spanish-speaking countries; Bermúdez-Aguirre, Mobbs, and Barbosa-Cánovas [20] showed that fresh cheeses made with ultrasonicated milk have better quality characteristics, better yield, and higher water-holding capacity. However, further research is needed to examine the potential of HIU treatment to aid cheese production. Their results were similar to those of the present study, since cheeses made with HIU milk retained more whey in the matrix.

### 3.2. CIE L*a*b* Color

Regarding the CIE L*, a*, and b* color parameters, significant differences were found in the tendency to red/green (a*, *p* = 0.0012), tendency to yellow/blue (b*, *p* = 0.0276), and hue angle (*p* < 0.0001) of Panela cheese due to the effect of HIU treatment time on fresh raw milk (Table 2). As HIU time increased, the value a* approached 0. Negative values in the color coordinate a* do not necessarily indicate a tendency towards green in the color space. Values closer to 0 indicate an achromatic tone (white), indicating higher whiteness in the Panela cheese. Consumers have a preference for the consumption of milk and fresh cheeses with higher whiteness; if HIU affects the color of the milk, it will probably produce the same effect on the products, as carotenoid constituents of milk are transferred into cheese with minimal losses and thus contribute to cheese coloration [25].

Similar results were obtained by Bermúdez Aguirre, Mawson, Versteeg, and Barbosa-Cánovas [26], who found that milk whiteness increased significantly as the intensity of HIU treatment increased. They observed significant changes in the values of L* and a*, which increased milk whiteness. These researchers also found negative values in a* (from −1.75 to −1.5) and positive values in b* (5.6–5.7). In the present study, the b* value and the hue angle were of greater relevance. Positive values in b* indicate a tendency towards yellow. The 10 min HIU treatment resulted in a higher b* value, presumably due to the carotenoids released during the breakdown of fat globules, these pigments being responsible for the yellow color in milk. This increase in yellowness due to ultrasound application might not be desirable in Panela cheese, so more research is still needed about the effects of HIU on Panela cheese color.

Accordingly, Ertugay, Sengül, and Sengúl [27] found that variations in the L*, a*, and b* parameters are due to a reduction in the size of the fat globule, which scatters the light, making the milk look whiter. As for the hue, according to the chromatic circle, the degrees obtained correspond to the yellow hue. The closest treatment at 90° (10 min HIU) produced a more yellow tone, while treatments higher than 100° (0 and 5 min HIU) produced a less yellow hue (from 90 to 120° the tone changed from yellow to green).

### 3.3. pH

**Figure 4:**
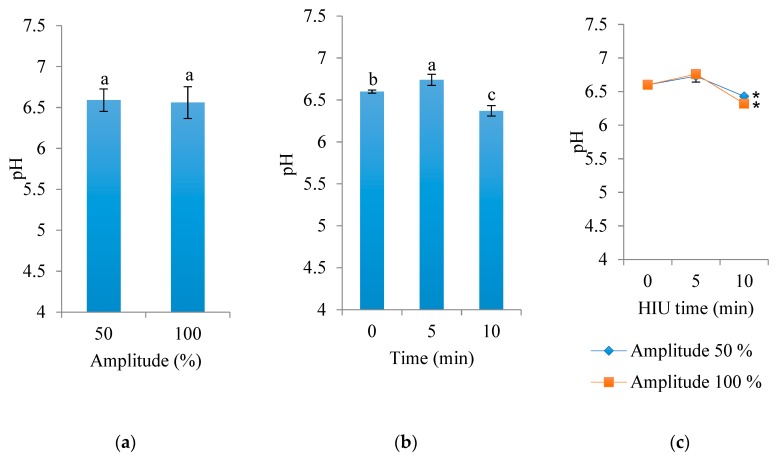
Effect of HIU amplitude on the pH of Panela cheese made with raw milk and stored for 24 h at 4 °C. (**a**) Effect of amplitude (mean of the two ultrasound times), (**b**) effect of time (mean of the two amplitudes), (**c**) interaction of both factors (time and amplitude). Asterisk (*) shows significant difference (*p* < 0.05) between treatments. ^a,b,c^ Different letters indicate significant differences between treatments (*p* < 0.05).

Statistical differences were found in the pH of Panela cheese due to the effect of time of HIU treatment (*p* < 0.0001). The interaction of HIU amplitude × time was also significant (*p* <0.0001). Cheese made with milk HIU-treated for a long time (10 min) had lower pH values (6.37) than cheese made with milk sonicated for short time (5 min). When using short HIU times, the cheese had a pH of 6.74; this value was closer to the fresh raw milk cheese. The controls had a pH of 6.6 (Figure 4). According to Jalilzadeh et al. [28], samples of Iranian feta cheese treated with frequencies of 20, 40, and 60 kHz and an intensity of 80% had lower pH values than the controls at the end of storage (60 d). It is worth mentioning that the pH values were in an acceptable range for Panela cheese, although the pH was reduced to 6.64.

HIU treatment time of 10 min and amplitude of 100% are perhaps sufficient to induce the cavitation phenomenon able to increase the enzyme activity of proteases or other native enzymes of milk [29]. In addition, there is evidence that ultrasound (24 kHz, 400 W) treatment can decrease the pH of fresh milk [24]. In a traditional process, the decrease in pH of mature cheeses during storage is due to the activity of lactic acid bacteria [29].

Several researchers have reported that the decrease in pH is due to the increase in the enzymatic activity on triglycerides due to the effect of cavitation, which, according to Wastra, Walstra, Wouters, and Geurts [30] and Uluko, Zhang, Liu, Tsakama, Lu, and Lv [31], produces the hydrolysis of phosphoric esters and the release of fatty acids into the medium. On the other hand, Supeno [32] demonstrated the formation of products such as nitrite, hydrogen peroxide, and nitrate in aqueous media by effect of ultrasound frequencies and intensities. On the other hand, other researchers have found no effect on the pH of milk treated with ultrasound. In this regard, Jambrak, Lelas, Mason, Krešić, and Badanjak [33] did not observe significant changes in serum protein pH due to the effect of low-intensity ultrasound treatment (20 kHz probe and 40 kHz bath) for 15 and 30 min. However, these researchers also reported the possible formation of hydroxyl and hydrogen peroxide free radicals due to cavitation.

### 3.4. Water, Protein, and Fat Contents

**Figure 5:**
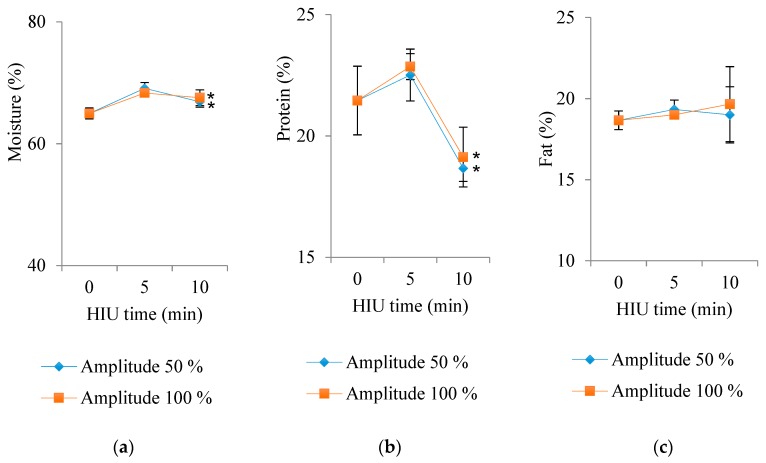
Effect of interaction of both factors (time and amplitude) of HIU on (**a**) moisture (%), (**b**) protein (%), and (**c**) fat (%) contents in Panela cheese made with HIU-treated raw milk and stored for 24 h at 4 °C. Asterisk (*) shows significant difference (*p* < 0.05) between treatments.

Regarding the moisture content (Figure 5), differences were found by effect of HIU time (*p* <0.0001), and the interaction between factors (amplitude × HIU time) was also significant (*p* = 0.0006). Panela cheese made with raw milk treated for 5 min with HIU had a higher moisture content (68.7%), which was significantly different from that of the controls (64.96%). The physicochemical properties of milk significantly affect the cheese yield and coagulation properties. It is well known that weak coagulation leads to higher protein losses, and consequently lower cheese yield and detrimental effects on the final texture of the product.

In this regard, Zhao, Zhang, Uluko, Liu, Lu, Xue, Kong, and Lv [13] reported increased denaturation of whey proteins and calcium and phosphorus contents by effect of ultrasound treatment (800 W, 0 and 20 min) in goat milk before coagulation with renin. Consequently, these researchers found that gelation characteristics such as firmness, strength (resistance), cohesiveness, and water retention capacity were significantly increased. Hence, ultrasound treatment produced highly branched structures with small uniform pores (such as honeycomb), while untreated milk produced pore sizes that were too large, with fewer interconnections. The increase in yield observed in the present study was mainly due to the higher moisture content. A partial contribution could have been related to other whey components ultimately retained in the casein matrix.

Regarding the protein content (Figure 5), significant differences were found between times of HIU (*p* = 0.0002). Those cheeses made with fresh raw milk treated for 0 and 5 min with HIU presented significantly higher protein contents (21.46% and 22.69%, respectively) than those made with milk treated ultrasonically for long periods (10 min), which presented an average of 18.9% of protein. Cheeses with high yield may not necessarily have been the highest in protein, since the higher yield could have been due to the presence of higher amounts of lactose and other solid whey compounds in the cheese. It is known that HIU improves the crystallization of ice and lactose in the cutting of cheese blocks [34], and this situation could have happened when the cheese was sonicated for the longer time of 10 min. Contrasting studies by Liu, Juliano, Williams, Niere, and Augustin [12] showed that ultrasound treatment induced structural changes in milk proteins. Chandrapala, Zisu, Kentish, and Ashokkumar [35] investigated the effect of ultrasonication (20 kHz, 31 W, 30 min) on the gelation of casein systems. Gels were formed by the addition of 7·6 mM Tetra Sodium Pyro Phosphate (TSPP) to 5 wt% micellar casein solutions and observed that sonication changed the surface hydrophobicity of the proteins, whereas surface charge was unaltered. This led to a firm gel formation with a fine network of protein and low syneresis. On the other hand, ultrasound treatment after the addition of the sodium salt produced a gel with weak structure and high syneresis.

Similar results were obtained in the present study when the milk was treated with HIU for 5 min, regardless of the amplitude. Obtained gels had higher water-holding capacity due to a more hydrophilic surface. On the other hand, Shanmugan and Ashokkumar [36] reported the formation of protein entities in lactic acid gels (milk 93% v/v and oil 7% v/v) treated with HIU. They found fine sonoemulsified oil globules stabilized with partially denatured whey proteins, which contributed to an improvement in the gel structure. Their observations might help to explain the findings of the present study where an increase in protein content was observed in cheese produced with raw milk treated with ultrasound for 5 min, regardless of the amplitude. In contrast, Villamiel and de Jong [37] and Barukčić, Jakopović, Herceg, Karlović, and Bozanić [38] observed a decrease in the whey protein content (alpha-lactalbumin and beta-lactoglobulin) in milk treated with continuous-flow ultrasound. However, this decrease was attributed to the denaturation caused by the thermosonication process in milk (61, 70, and 75.5 °C). No changes in milk casein were observed under the experimental conditions analyzed in the present study. Hence, it was considered that the increase in protein content could have been due to the increase in whey protein content in the phosphocasein matrix (cheese) of ultrasound-treated milk, compared to untreated controls. Jalilzadeh, Hesari, Peighambardoust, and Javidipour [28] found no differences in the protein content of Iranian feta cheese matured for 60 d, made from ultra-filtered pasteurized milk and ultrasonicated (20, 40 and 60 kHz, 80% intensity, 20 min). However, they found high rates of lipolysis (free fatty acid content) and proteolysis (water-soluble nitrogen).

Regarding fat content (Figure 5), no differences were found by effect of any factor (*p* = 0.85). The fat content in cheese made with HIU-treated milk ranged between 18.66% and 19.33%. Shanmugan and Ashokkumar [36] found a decrease in the syneresis of acid gels (milk and linseed oil) of up to 71% when they were ultrasonicated (20 kHz, 176 W, 1–8 min). Protein entities associated to fat (fat globules whose surface had denatured whey proteins) have a significant role in water retention, acting as fillers by forming crosslinks with other proteins to retain water molecules in the gel network [39]. Thus, sonoemulsified flaxseed oil milk gels are protein gels with small pores and improved water retention capacity, while pasteurized homogenized skim milk gels produced large pore structures with high syneresis.

Villamiel and de Jong [37] reported a substantial reduction (up to 81.5%) in the size of ultrasound-treated milk fat globules, with no changes in fat content compared to controls. Bermudez-Aguirre, Mawson, Versteeg, and Barbosa-Cánovas [26] also found an increase in fat content and yield, as a positive effect on the thermostability of cream cheese made from thermosonicated milk. They reported a reduction in the size of the fat globules, as well as their roughness and disruption.

In accordance with the results of the present study, Jalilzadeh, Hesari, Peighambardoust, and Javidipour [28] found no differences in the fat content of Iranian feta cheese made with ultrafiltered milk pasteurized and treated with ultrasound (20, 40 and 60 kHz, intensity 80%, 20 min) after 60 d of maturation.

### 3.5. Microbial Counts

**Figure 6:**
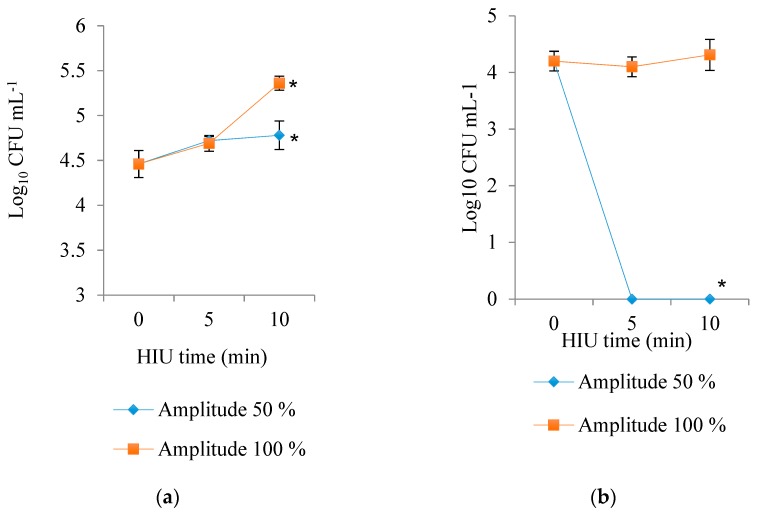
Effect of the interaction of both factors (time and amplitude) of HIU on mesophilic (**a**) and coliform (**b**) bacteria counts of Panela cheese made with HIU-treated raw milk and stored for 24 h at 4 °C. Asterisk (*) shows significant difference (*p* < 0.05) between treatments.

HIU amplitude (*p* = 0.0074) and time (*p* < 0.0001) showed significant effect on the growth of mesophilic bacteria present in Panela cheese (Figure 6). The use of 50% amplitude significantly decreased mesophilic bacterial counts from 4.83 log (100% amplitude) to 4.65 log, while an increase of HIU time significantly increased mesophilic counts from 4.46 log (control) to 5.07 log (10 min HIU). A change in growth phase was observed, probably from lag to log phase.

The controls had less total aerobic load, while the products treated with HIU showed an increase in the counts of mesophilic bacteria. Increase of bacterial counts after a period of ultrasonication may be multifactorial and it might be caused by the release of nutrients from meat subjected to cavitation [40]. Additionally, ultrasound may increase the rate of transport of oxygen and nutrients into bacterial cells, enhancing their growth [41]. Therefore, we could argue that the lower pH in cheeses ultrasonicated for a longer time (10 min), a higher growth of lactic-acid-producing bacteria occurred.

Herceg, Juraga, Sobota-Šalamon, and Rezek-Jambrak [42] reported significant inactivation of mesophilic bacteria in fresh raw milk upon long periods of ultrasonication treatment in combination with temperature and amplitude. The lowest counts (3.99 log CFU/mL) were obtained with amplitude of 120 μm, temperature of 60 °C, and 12 min treatment. However, when the ultrasonication temperature was maintained at 20 °C, mesophilic bacterial counts were not reduced after ultrasound treatment, similarly to the present study. In fact, when Herceg, Juraga, Sobota-Šalamon, and Rezek-Jambrak [42] increased the amplitude to 120 μm, mesophilic counts increased significantly from 6.34 (untreated fresh raw milk) to 7.86 log CFU/ml (120 μm, 20 °C, 6 min). This microbial inactivation was attributed to temperature change. Ganesan, Martini, Solorio, and Walsh [43] also found that microbial load in milk could be significantly reduced by up to 5 log when thermosonication conditions were 72 °C, 10 s, and 216 μm. On the other hand, mesophilic bacteria in milk include both Gram-positive and Gram-negative groups, and the sensitivity of these groups to ultrasound treatment is still controversial [44,45,46].

HIU treatment using amplitudes of 50% (*p* < 0.0001) was effective in decontaminating coliform bacteria (Figure 6), regardless the application time (5 or 10 min). While the use of 100% amplitude did not decrease the counts of coliform bacteria, neither did the microbial load increase with short or long HIU treatment time. The results of the present study, with an amplitude of 50%, regardless of HIU time, are similar to those obtained by Jalilzadeh, Hesari, Peighambardoust, and Javidipour [28]. They found that Iranian feta-type cheese produced from ultrafiltered, pasteurized milk and inoculated with *Escherichia coli*, when treated with ultrasound (20, 40, and 60 kHz, 80% intensity), had lower counts of *E. coli* than cheese (60 days of maturation) made with untreated milk.

On the other hand, Cameron, McMaster, and Britz [47] reported the capacity of ultrasound (20 kHz, 750 W) to decrease pathogens and deterioration bacteria in milk. They showed that viable *E. coli* cell counts were reduced by 100% after 10 min of ultrasound treatment. Likewise, other bacteria such as *Pseudomonas fluorescens* and *Listeria monocytogenes* were also reduced by 100% and 99% after 6 and 10 min of treatment, respectively. In the present study, psychrophilic bacteria counts were zero in all the treated cheeses, most probably due to the short storage period (24 h) at 4 °C. However, we cannot exclude that an increase in psychrophilic bacteria might have taken place after longer storage of the cheese, as demonstrated by Sams and Feria [40] and Pitt and Ross [41].

Long storage times should increase the counts of psychrophilic bacteria in Panela cheese made with ultrasonicated raw milk. Many researchers have reported that the ultrasonication process alone has little bacterial inactivation power at room temperature [48]. Hence, the latest research has focused on the use of assisting technologies to ultrasound, such as temperature (thermosonocation) and pressure (mannosication). In this regard, Barukčić, Jakopović, Herceg, Karlović, and Bozanić [38] reported that an increase of temperature from 45 to 55 °C, together with ultrasonication (480 and 600 W), contributed to an increase of inactivation (from 1 to 2 log cycles) of bacteria responsible for deterioration in reconstituted milk. Subsequently, Noci, Walkling-Ribeiro, Cronin, Morgan, and Lyng [49] found that short ultrasonic times (160 s) at 25 °C produced a reduction of only 1.2 log cycles of harmless *Listeria* spp. in milk.

## 4. Conclusions

The ultrasonication of fresh raw milk significantly increased the yield of Panela cheese. The highest yield was observed when milk was ultrasonicated for 10 min, regardless of the amplitude of sonication. As the ultrasonication time increased, the b* value increased and the hue angle significantly decreased. Therefore, the cheeses treated for 5 and 10 min showed a more yellow tone than the controls. The pH had an unequal behavior, increasing significantly from 6.6 (control) to 6.74 (5 min HIU) and decreasing to 6.37 when the milk was treated for 10 min, probably due to the growth of lactic acid bacteria. Fat was not affected by HIU, but the protein content increased when the milk was treated with HIU for 5 min. Although HIU was not an efficient technology for the control of mesophilic bacteria, coliform bacteria were completely inactivated in Panela cheeses when the milk was ultrasonicated with 50% amplitude. Ultrasound treatment could be a valid tool to improve the cheese yield and microbiological quality of Panela cheese made with fresh raw milk.

## Figures and Tables

**Table 1:** Physicochemical parameters of the fresh raw milk.

Titratable Acidity (°D)	Fat (%)	Protein (%)	Lactose (%)	Non-Fatty Solids (%)
16	3.87	3.11	4.79	8.6

**Table 2:** Factorial effect of amplitude and sonication time on L*, a*, and b* (mean value ± standard deviation) parameters of Panela cheese.

Factor	CIE Space L*, a*, and b*				
Amplitude (%)	Luminosity (L*)	a*	b*	Hue	Chrome
**50**	92.74 ± 2.77 ^a^	−2.27 ± 1.92 ^a^	11.37 ± 1.21 ^a^	101.4 ± 1.46 ^a^	11.6 ± 2.21 ^a^
**100**	92.4 ± 3.89 ^a^	−2.32 ± 1.61 ^a^	11.76 ± 1.13 ^a^	101.1 ± 2.27 ^a^	12.0 ± 1.82 ^a^
**Sonication time (min)**					
**0**	92.49 ± 3.55 ^a^	−2.51 ± 1.76 ^b^	10.77 ± 1.31 ^b^	103.1 ± 2.10 ^a^	11.06 ± 2.08 ^a^
**5**	92.48 ± 3.13 ^a^	−2.63 ± 1.94 ^b^	11.68 ± 1.31 ^b^	102.6 ± 2.43 ^a^	11.97 ± 2.20 ^a^
**10**	92.75 ± 3.13 ^a^	−1.75 ± 1.94 ^a^	12.25 ± 1.31 ^a^	98.07 ± 2.43 ^b^	12.38 ± 2.20 ^a^

^a,b^ Different letters within the same row indicate significant differences between treatments (*p* < 0.05).
